# The Wandering Circles: A Flicker Rate and Contour-Dependent Motion Illusion

**DOI:** 10.1177/2041669519875156

**Published:** 2019-09-25

**Authors:** Christopher D. Blair, Gennady Erlikhman, Gideon P. Caplovitz

**Affiliations:** Department of Psychology, Eastern Oregon University, La Grande, OR, USA; Department of Psychology, University of Nevada, Reno, NV, USA; Department of Psychology, University of California, Los Angeles, CA, USA; Department of Psychology, University of Nevada, Reno, NV, USA

**Keywords:** motion, perception, visual illusion, flicker

## Abstract

Understanding of the visual system can be informed by examining errors in perception. We present a novel illusion—Wandering Circles—in which stationary circles undergoing contrast-polarity reversals (i.e., flicker), when viewed peripherally, appear to move about in a random fashion. In two psychophysical experiments, participants rated the strength of perceived illusory motion under varying stimulus conditions. The illusory motion percept was strongest when the circle’s edge was defined by a light/dark alternation and when the edge faded smoothly to the background gray (i.e., a circular arrangement of the Craik-O’Brien-Cornsweet illusion). In addition, the percept of illusory motion is flicker rate dependent, appearing strongest when the circles reversed polarity 9.44 times per second and weakest at 1.98 times per second. The Wandering Circles differ from many other classic motion illusions as the light/dark alternation is perfectly balanced in time and position around the edges of the circle, and thus, there is no net directional local or global motion energy in the stimulus. The perceived motion may instead rely on factors internal to the viewer such as top-down influences, asymmetries in luminance and motion perception across the retina, adaptation combined with positional uncertainty due to peripheral viewing, eye movements, or low contrast edges.

The ability to accurately see the motion of objects around us is an essential component of visual perception. Here, we introduce a novel visual illusion that we call the Wandering Circles. The Wandering Circles is a dynamic illusion in which stationary objects appear to move. The illusion manifests when simple objects (i.e., circles) undergo contrast-polarity reversals and are viewed peripherally. While we would like to be able to state that we discovered this illusion through theoretically driven stimulus design, we first noticed the illusion quite by accident while developing stimuli for another completely unrelated project. In the configuration highlighted in Figure 1 and viewable in Demonstration Video 1, the circles are defined by contours that induce the Craik-O’Brian-Cornsweet Illusion (Cornsweet circles). In the video, the contrast polarity of these contours reverses ∼10 times a second (e.g., roughly 100 ms passed before each polarity change). When an observer maintains fixation near the center of this display, after a brief period of time, the circles will appear to randomly move around (i.e., wander). Viewers differ in their perceived strength of the illusion, as well as in how long it takes for the illusion to manifest. We have found that the illusion is strongest if stimuli are viewed peripherally, with the gaze held in a relaxed fashion. Also, the illusion often emerges over time, and the viewer should give themselves a few seconds of relaxed peripheral viewing before the illusion may manifest. At times, these illusory motions can be quite striking, with the circles appearing to follow remarkably long trajectories. Sometimes the circles will appear to follow the same trajectory, all moving together as a single group. At other times, each circle can appear to follow its own meandering path. This latter observation of independent motion trajectories demonstrates that the phenomenon is not solely caused by motion signals across the retina generated by the movements of the eyes. Because eye movements are almost exclusively translational in nature (Schütz, Braun, & Gegenfurtner, 2011), such eye movement-related motion signals would not be expected to generate percepts of independent motion for each of the circles.

**Figure 1.**
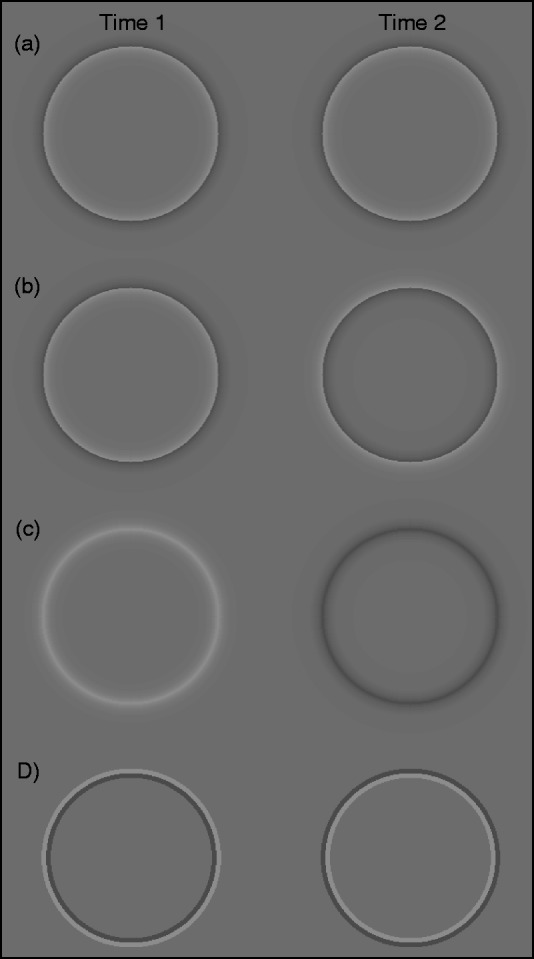
Stimuli used in Experiments 1 and 2. (a) Cornsweet circles: nonflickering Cornsweet circles; (b) flickering Cornsweet circles: Cornsweet circles reversing polarity over time; (c) unipolar Gaussians: circles with only a light or a dark edge fading to the color of the background that change between light and dark gray over time; and (d) solid lines: circles with a light and dark gray line at their edge that exchange positions over time.

**Video 1.** Demonstration of the typical Wandering Circles illusion with circles reversing polarity at a rate of 9.44 times per second.

The Wandering Circles join a class of illusions in which motion is either seen when none is present, or when the perceived motion is different than that physically present in the image. The Wandering Circles illusion appears to share many features with previously examined motion illusions such as ambiguity in the perceived motion (Anstis & Ramachandran, 1986), as well as the necessity of flicker (Anstis & Rogers, 1975, 1986; Mather, 2017), peripheral viewing (De Valois & De Valois, 1991; Ramachandran & Anstis, 1990), and destabilized eye movements (Beer, Heckel, & Greenlee, 2008). However, as will be discussed further in the general discussion, in spite of these similarities, the specific combination of features and resulting percept of the Wandering Circles is unique when compared with other motion illusions such as apparent motion, reversed-phi motion, and drifting-Gabors (Anstis & Ramachandran, 1986; Anstis & Rogers, 1975, 1986; De Valois & De Valois, 1991; Mather, 2017).

Before attempting to assess what the Wandering Circles may clarify about the functioning of motion perception, it is necessary to first determine the stimulus conditions under which the Wandering Circles are most readily observed. Here, we describe the results of two experiments that probe two such factors: flicker rate and type of circle-defining contour that, in our initial observations, appear to be critically important for the manifestation of the illusion. Using a magnitude-estimation paradigm, we find that both flicker rate and contour-type modulate the strength of the illusion.

## Experiment 1

### Methods

#### Participants

Note that participant demographics apply to participants from both Experiments 1 and 2. Participants consisted of undergraduate student volunteers from the University of Nevada, Reno and an associate professor at the University of Nevada, Reno. There were a total of 24 participants with 10 of those participating in both Experiments 1 and 2. Of those 24, 17 participants reported demographic information. Of these, 10 identified as female, 6 as male, and 1 as other. The mean reported age was 24.4 years (*SD* = 6.8). Participants in Experiment 1 (*n *=* *17) were naïve to the specific aims of the study before completing the experiment. All reported having normal or corrected-to-normal vision.

#### Presentation

Stimuli were presented on a Mitsubishi DiamondPro 2070 SB monitor (22-in., 1,024 × 768 pixel resolution) with an 85-Hz refresh rate. The stimulus computer was a 2.5-GHz Mac Mini with an Intel HD Graphics 4000 768 MB graphics processor (16 GB of DDR3 SDRAM). Stimuli were created and presented with the Psychophysics Toolbox (Brainard, 1997; Pelli, 1997) for MATLAB (version 2017b; Mathworks Inc., Natick, MA). Participants viewed the stimuli binocularly from a distance of 57 cm with their chin positioned in a chin rest. Eye movements were not monitored.

#### Stimuli

Two of the most readily apparent physical aspects of the Cornsweet circles stimulus in which we first observed the illusion are the abutting lighter and darker areas, as well as the Gaussian fading of the stimulus into the background. Thus, we developed three distinct stimuli for this experiment, each distinguished by the way their contours were defined. Cornsweet circles, as shown in Figure 1(a) and 1(b), had contours defined by a light gray (41 cd/m^2^) and dark gray (16 cd/m^2^) edge smoothly changing to the background gray (27 cd/m^2^) in an exponential fashion. Gaussian circles, as shown in Figure 1(c), had contours that were either light gray or dark gray and faded to the background gray both inward and outward in an exponential fashion. Finally, we examined solid-line circles that had contours defined by abutting light and dark gray lines, as shown in Figure 1(d). Each circle had a radius of 2° as measured from the center of the circle to the center of its contour.

#### Procedure

##### Passive example viewing

Before beginning the actual experiment, participants first viewed three examples of how three different levels of real motion might relate to a 1 to 9 Likert scale. They first viewed a nonflickering stationary Cornsweet circle at the center of the screen for 5,000 ms, while the label *Motion Level 1* was displayed below it. Participants then viewed a Cornsweet circle that moved in a random direction up to 0.15°, roughly 10 times a second (9.44 times), thus corresponding to a speed of up to 1.5°/second within a constraint of 1° vertically or horizontally from the center of the screen, while the label *Motion Level 5* was displayed below it. Finally, participants viewed a Cornsweet circle that moved in a random direction up to 0.3°, 10 times a second, thus corresponding to a speed of up to 3°/second. The label *Motion Level 9* was displayed below it. Circles would start their motion in a randomly chosen direction and reverse their vertical or horizontal direction whenever they reached the limits of their motion constraint.

##### Active practice

**Figure 2.**
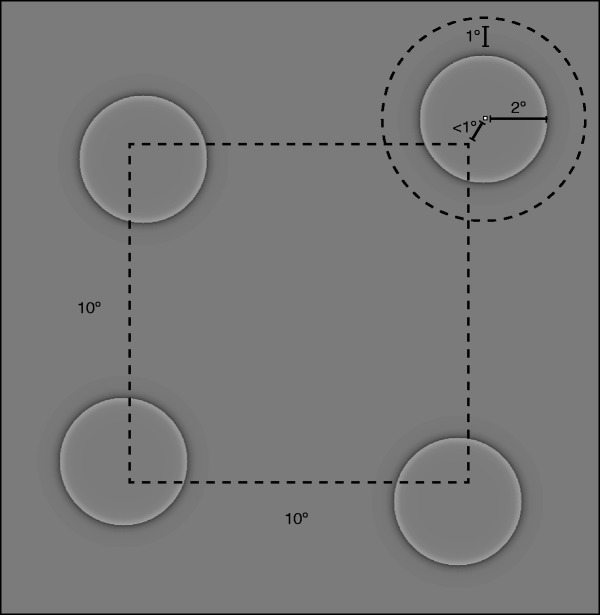
Stimulus arrangement. On each trial, four stimulus circles were displayed in a roughly square arrangement, each with a starting position no further than 1° from the corner of a 10° square. On trials in which there was real motion, stimuli moved no further than 1° from their original starting location before changing direction.

After viewing the three examples, participants were then presented with seven practice trials. On each of these practice trials, as illustrated in Figure 2, four Cornsweet circles were presented in a roughly square shaped pattern (10° by 10°) centered on the screen center. The starting location for each circle could vary by up to 1° vertically or horizontally from the respective corner of the square pattern. As in the Passive example trials, the four Circles would start their motion in a randomly chosen direction and reverse their vertical or horizontal direction whenever they reached the limits of their 1° motion constraint. A different level of motion was shown on each 5,000 ms trial: up to a displacement of 0°, 0.03°, 0.09°, 0.15°, 0.21°, 0.27°, 0.3° per step, at a rate of 9.44 steps per second (see Demonstration Video 2). After the circles disappeared, participants were instructed to indicate, on a 1 to 9 Likert scale, how much motion they perceived.

**Video 2.** Demonstration of real motion condition in which circles change physical position at a rate of 9.44 times per second.

##### Experimental trials

The experimental procedure was identical to that used during the active practice, with the exception that participants completed 168 trials, which included 42 trials in which stimuli were Cornsweet circles moving at one of the previously listed speeds (6 trials at each speed), 42 trials in which stimuli were stationary Cornsweet circles reversing polarity 9.44 times a second (see Demonstration Video 1), 42 trials in which stimuli were stationary unipolar Gaussian circles reversing polarity 9.44 times a second (see Demonstration Video 3), and 42 trials in which stimuli were stationary solid-line circles reversing polarity 9.44 times a second (see Demonstration Video 4).

**Video 3.** Demonstration of the unipolar Gaussian condition used in Experiment 1 with circles reversing polarity at a rate of 9.44 times per second.

**Video 4.** Demonstration of the solid-line condition used in Experiment 1 with circles reversing polarity at a rate of 9.44 times per second.

##### Analysis

**Figure 3.**
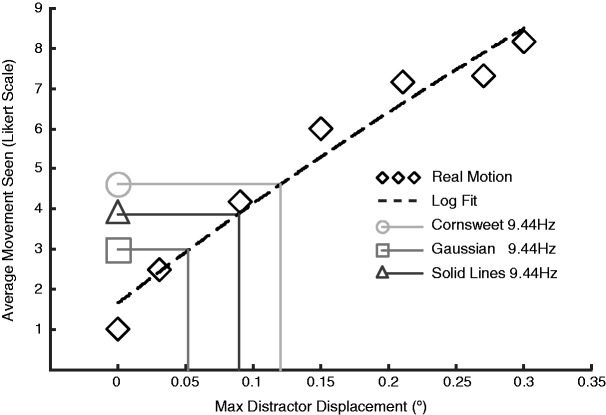
Estimating illusion magnitude. Representative data from one participant. Likert ratings in response to real motion (diamonds) were modeled using logarithmic regression (dashed line). This model was then used to estimate the physical equivalents of the perceived motion in the 9.44 reversals per second flickering stimuli for the Cornsweet (circle), solid-line (triangle), and Gaussian (square) conditions. For this typical participant, there is a strong positive relationship between the physical motion and reported motion. Although they were not physically moving, the participant rated the 9.44 reversals per second flickering Cornsweet circle, on average, at 4.6 on the Likert scale, which according to the logarithmic model, corresponds to a physical displacement of ∼0.12° per step or 1.2°/second. In contrast, for this participant, the solid lines exhibited less illusory motion and the Gaussians even less.

Likert ratings of real motion stimuli served two purposes in this study to contextualize the Likert scale ratings given by participants for the flickering, physically nonmoving stimuli (e.g., how do ratings for illusory motion compare with ratings for real motion; Figure 3). A relationship was modeled with a logarithmic function (*y *=* a* × ln(*x*) + *b*) correlating the real motion and Likert scale ratings for each participant (see Figure 3). A log fit was chosen as it was shown to have a closer fit than a linear fit, as measured by *R*^2^ values, for the majority of the participants’ data. Thus, for each subject, we were able to obtain both a raw Likert rating and a value corresponding to the estimated strength of the illusion in terms of real motion for each of the three flickering stationary stimulus conditions. At the group level, we ran a Friedman test, as well as Wilcoxon Signed-rank tests to evaluate the statistical significance of any differences in perceived illusion strength between the three flickering stationary stimulus conditions. Nonparametric statistical methods were employed to account for potential violations of the necessary assumptions due to the use of Likert scale ratings as the primary measure. That being said, patterns of significant and nonsignificant results were identical when parametric equivalents were used to analyze the data.

### Results

**Figure 4.**
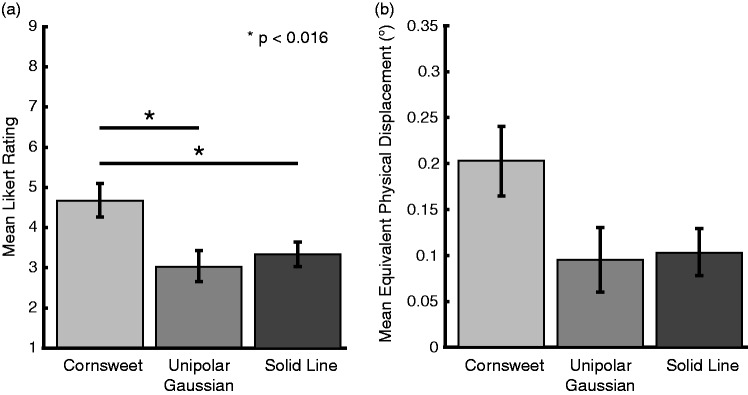
Experiment 1 results: (a) mean Likert ratings for amount of motion observed for 9.44 reversals per second flickering Cornsweet, unipolar Gaussian, and solid-line circles. At the group level, Cornsweet circles were perceived to have a greater illusion strength than either other stimulus type. It should be further noted that in spite of the lack of a significant difference in the perceived illusion strengths for the Gaussian and solid-line conditions, of the individual participants, only four produced an average illusion strength score that was higher for Gaussian circles than the solid-line stimuli. (b) Mean amount of motion observed in each condition in terms of real motion calculated using the log fit of real motion and Likert ratings for nonflickering Cornsweet circles. Error bars represent standard error.

**Figure 5.**
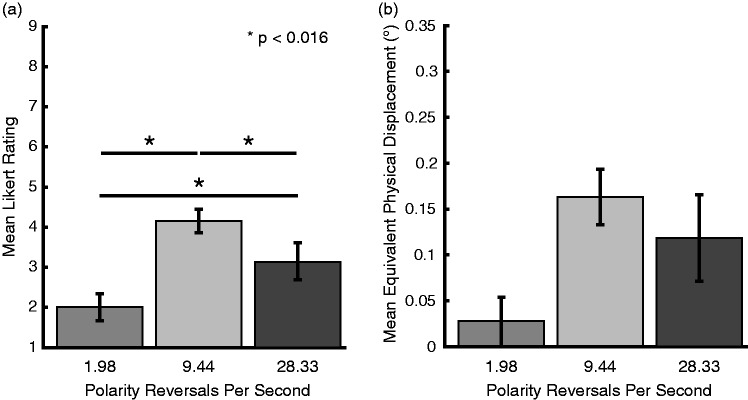
Experiment 2 results: (a) mean Likert ratings for amount of motion observed for 1.98, 9.44, and 28.33 reversals per second flickering Cornsweet circles; 9.44 and 28.33 reversals per second flickering circles both produced motion percepts greater than 1.98 reversals per second flickering circles, and the 9.44 reversals per second flickering circles also produced an illusion strength greater than that produced by the 28.33 reversals per second flickering circles. (b) Mean amount of motion observed in each condition in terms of real motion calculated using the log fit of real motion and Likert ratings for nonflickering Cornsweet circles. Error bars represent standard error.

Using average Likert scale ratings, we performed a Friedman test to determine if there were significant differences in the perceived motion for each stimulus type overall, as well as Wilcoxon Signed-rank tests to determine whether there was a significant difference in the illusion strength created by each flickering stimulus. The results of the Friedman test were compared against an alpha level of .05. As shown in Figure 4(a), there were significant differences in the strengths of the illusion perceived across stimulus types, χ^2^(2) = 24.471, *p* < .001. As demonstrated in post hoc comparisons (Bonferroni-adjusted critical *p =* .016), the Cornsweet circles produced an illusion strength that was significantly greater than that for both the unipolar Gaussians (*Z *=* *3.621, *p* < .001, *r* = .878) and the solid lines (*Z *=* *3.574, *p* < .001, *r* = .867). However, there was no significant difference in the illusion strength between the unipolar Gaussians and solid lines (*Z *=* *1.942, *p* = .052, *r* = .471; Figure 4(a)). Further, we show that when real motion equivalents are calculated for each condition use the log fits, a similar pattern is observed across the Cornsweet circles (*M *=* *0.202°, *SD* = 0.157°), unipolar Gaussians (*M *=* *0.095°, *SD* = 0.145°), and solid lines (*M *=* *0.104°, *SD* = 0.104°) conditions (Figure 5(b)).

## Experiment 2

### Methods

#### Participants

Participants in Experiment 2 (*n *=* *17) were also naïve to the specific aims of the study before completing the experiment. However, participants were informed as to the existence of the illusion and had the illusion demonstrated to them before beginning Experiment 2.

#### Stimuli

Stimuli in Experiment 2 consisted of the same Cornsweet circles used in Experiment 1, both the 9.44 reversals per second polarity reversing, and nonpolarity reversing, with the addition of Cornsweet circles reversing polarity at 1.98 times a second (see Demonstration Video 5) and at 28.33 times a second (see Demonstration Video 6). As our initial observation of the Wandering Circles illusion was made with a 9.44 reversals per second flickering stimulus, these particular flicker rates were chosen to determine whether the effect is specific to 9.44 reversals per second or whether it can also be observed at significantly higher or lower rates.

**Video 5.** Demonstration of one condition used in Experiment 2 with Cornsweet circles reversing polarity at a rate of 1.98 times per second.

**Video 6.** Demonstration of one condition used in Experiment 2 with Cornsweet circles reversing polarity at a rate of 28.33 times per second.

#### Procedure/analysis

The methods and analysis used in Experiment 2 were identical to those used in Experiment 1, with the exception that the experimental manipulation here was flicker rate: 1.98, 9.44, and 28.33 reversals per second.

### Results

The results of a Friedman test examining the Likert scale ratings showed that there were again significant differences in the strength of the illusion across the different stimulus frequencies, χ^2^(2) = 25.529, *p* < .001. Again, results of the Friedman test were compared against an alpha level of .05. As demonstrated in post hoc comparisons (Bonferroni-adjusted critical *p =* .016), Wilcoxon Signed-rank tests demonstrated that the 9.44 reversals per second circles were perceived as having a significantly greater illusion strength than the 1.98 reversals per second circles (*Z *=* *3.621, *p* < .001, *r* = .878) and 28.33 reversals per second circles (*Z *=* *2.864, *p* < .001, *r* = .695), and the 28.33 reversals per second circles were also perceived as having a significantly greater illusion strength than the 1.98 reversals per second circles (*Z *=* *3.574, *p* < .001, *r* = .867; Figure 5(a)). We again show that when real motion equivalents are calculated for each condition use the log fits, a similar pattern is observed across the 1.98 reversals per second (*M *=* *0.027°, *SD* = 0.111°), 9.44 reversals per second (*M *=* *0.164°, *SD* = 0.124°), and 28.33 reversals per second (*M *=* *0.118°, *SD* = 0.196°) circles conditions (Figure 5(b)).

We also note that the 9.44 reversals per second condition tested in this experiment used the same physical stimulus as that used in the Cornsweet circle condition in Experiment 1. A Mann–Whitney test revealed no significant difference between the average Likert scale ratings obtained for these two sets of data (*U = *118*, p =* .361, *r* = .157), thus demonstrating an internal replication of our results across two groups of participants. Importantly, these groups differed not only in the other stimulus conditions (contour differences vs. frequency differences) they experienced but also in the fact that the participants in Experiment 1 were not informed that they would be viewing illusory motion, when compared with the viewers in Experiment 2 who had the illusion demonstrated to them in advance. The comparable results for both groups suggest that knowledge of the illusion is neither necessary to perceive motion nor is it sufficient to disrupt the illusory motion percept.

## Discussion

We introduced a novel visual illusion in which flickering stationary circles appear to move. Our empirical findings suggest an important role for how the contours are defined in that we found the strongest effects when the circles were defined by our Cornsweet edges. This is likely to be the case because as the contrast-polarity switches, there are local directional spatiotemporal correspondences between the lighter and darker contour regions signaling opposing inward and outward motion, as well as a potential increase in positional uncertainty created by the gradual fade of the edge color to that of the background. The correspondences that minimize the spatial displacement in a point-by-point manner will be orthogonal to the circular contour and be equivalent to what would be expected to be detected by an aperture-problem-constrained motion sensitive neuron (Pack & Born, 2001; Pack, Livingstone, Duffy, & Born, 2003). In contrast, the unipolar edges, which generated the weakest illusion percept, do not contain this correspondence specifying the local directional motion signal but instead creates a general motion impulse with no locally or globally specified direction. Like the Cornsweet edges, the solid-line configuration does contain local inward–outward directional correspondences; however, the spatial displacement is overall less than in the Cornsweet case and lacks the additional positional uncertainty associated with a Gaussian edge. This likely accounts for why the observed magnitude of the solid-line case was intermediate to the Cornsweet and unipolar conditions. We also found that the illusion was most readily observed at 9.44 reversals per second, followed by 28.33 reversals per second, whereas at 1.98 reversals per second, the illusion was much weaker, indicating that a minimum amount of flicker may be necessary to induce the illusion. This finding may be related to a similar phenomenon observed in which stimuli flickering at faster rates were shown to suppress the perception of stimuli flickering at lower rates with which they were overlapped (Cass & Alais, 2006). Specifically, stimuli needed to flicker at a speed of at least 6 Hz to produce this perceptual suppression. However, while not empirically tested here, one can see in looking at additional demonstrations using a variety of refresh rates (Video 7): ∼4, Video 8: ∼6, Video 9: ∼15, Video 10: ∼45, and Video 11: ∼60 reversals per second) that there is still the possibility to see illusory motion at rates 6 Hz and lower and that the stimuli themselves become less visible at higher frequencies. However, additional research with flickering stimuli has demonstrated that only reversal frequencies above ∼6 reversals a second impaired an observer’s accuracy in identifying real and illusory motion when reversal speeds of 1.98, 3.86, 5.67, 6.54, 7.73, 8.50, 9.44, 10.62, or 12.14 were tested (Erlikhman, Gutentag, Blair, & Caplovitz, 2019).

**Video 7.** Demonstration of Wandering Circles illusion with circles reversing polarity at a rate of roughly 4 times per second.

**Video 8.** Demonstration of Wandering Circles illusion with circles reversing polarity at a rate of roughly 6 times per second.

**Video 9.** Demonstration of Wandering Circles illusion with circles reversing polarity at a rate of roughly 15 times per second.

**Video 10.** Demonstration of Wandering Circles illusion with circles reversing polarity at a rate of roughly 45 times per second.

**Video 11.** Demonstration of Wandering Circles illusion with circles reversing polarity at a rate of roughly 60 times per second.

Though not empirically tested here, we offer a few additional observations of particular note associated with the Wandering Circles illusion. First, pilot testing showed that the illusory motion percept appeared weaker when strict fixation was maintained Video 12). This is in line with studies showing that the strength of peripheral drift illusions can be increased when eye movements are destabilized (Beer et al., 2008). However, it should be reemphasized that when multiple wandering circles are viewed simultaneously, they often appear to move independently of one another, which makes it unlikely that the apparent motion of these stimuli is entirely reliant on the particular direction in which an observer’s eyes move. Second, we note that the strength of the Wandering Circles illusion also depends on eccentricity. As can be seen in Demonstration Video 1(Figure 8), if one of the circles is fixated, it tends not to appear to move, whereas the more peripheral ones do. It may also be noted that the seemingly omnidirectional random motion pattern is, to some degree, reliant on the contour of the flickering shape, as when a similar stimulus is created using straight lines (Video 13), the directions of perceived motion appear to be constrained to those perpendicular to the line itself. This may be unsurprising, as in the case of the circles, there is local motion in all directions simultaneously, but only in the two directions orthogonal to the contour in the case of the lines. And finally, one may also note that the stimuli used in each of our experiments and demos flicker in phase with one another. However, further observations indicate that this is not necessary to produce the illusory motion percept as demonstrated in Video 14 where circles flicker at the same frequency, but out of phase with one another.

**Video 12.** Demonstration of typical Wandering Circles illusion but with a central fixation point present. One may note that the strength of the illusion is reduced when strict fixation is maintained.

**Video 13.** Demonstration with circles replaced by Cornsweet lines reversing polarity. One may note that perceived motion is constrained to be perpendicular to the lines.

**Video 14.** Demonstration of the typical Wandering Circles illusion, but with circles flickering out of phase with one another, but still at the same frequency. One may note that this does not appear to affect the overall strength of the illusion.

As previously stated, the Wandering Circles illusion joins a large number of other illusions in which stationary objects appear to move or have their perceived positions influenced by motion. One class of these illusions is best highlighted with so-called drifting-Gabor stimuli. In such illusions, the internal motion of the object leads to an illusory perception of the object’s overall motion (De Valois & De Valois, 1991; Lisi & Cavanagh, 2015; Morgan, 2017; Ramachandran & Anstis, 1990; Tse & Hsieh, 2006; Whitney et al., 2003). Similar to such illusions, the strength of the Wandering Circles illusion tends to be much stronger in the periphery than at fixation. This is likely due to a combination of spatial resolution and cortical magnification that allows a much more precise coding of location at fixation than in the periphery, thus allowing motion signals to bias perceived position. Another class of motion illusions that resemble the Wandering Circles in their use of flicker in producing an illusory motion percept is reversed-phi, two-stroke, four-stroke apparent motion and the recently described perceptual diamond (Anstis & Rogers, 1975, 1986; Flynn & Shapiro, 2018; Mather, 2017). However, in each of these cases, and for drifting-Gabor stimuli, in contrast to the Wandering Circles, there is an implied direction for the illusory motion that leads to an illusory motion percept in that particular direction, suggesting that the Wandering Circles illusion is caused, at least in part, by some previously untested characteristic.

What appears to truly set the Wandering Circles apart is the equal and opposite local motion energy, which sums to no net global motion that only exists at the level of the image. In principle, there are many sources of variability that can make this image-level lack of net motion be perceived as net directional motion in the brain. Examples of such sources include heterogeneity in the sensitivity and selectivity of motion-detecting neurons across the visual field (Born & Bradley, 2005), heterogeneity in the rate and strength of adaptation of those neurons, as well as intrinsic neural noise along the motion processing hierarchy (Shadlen, Bitten, Newsome, & Movshon, 1996), the effects of each of these being amplified by small fixational eye movements. Thus, when these, and potentially many other, sources of variability are taken together, there likely are directional motion signals in the Wandering Circles at the level of the brain.

**Figure 6.**
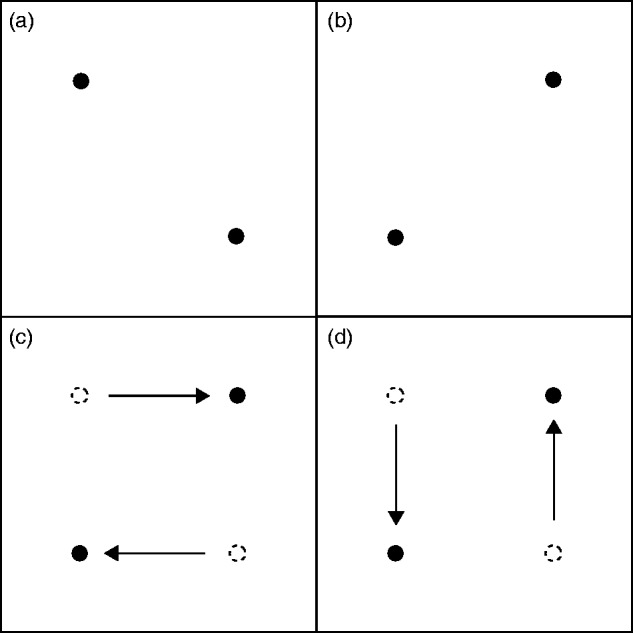
Apparent motion display. A two-frame display in which two dots occupy positions at the top left and bottom right points of an imaginary square at Time 1 (a) and the top right and bottom left points Time 2 (b). Observers generally report that the dots appear to move between the two positions rather than simply appearing. At each position change, dots may be observed to move consistent with the interpretation presented in (c) or that in (d).

This dichotomy between no net global motion in the image and directional motion in the brain is reminiscent of stimuli such as ambiguous dot-quartet apparent motion displays like those shown in Figure 6. However, while such stimuli do exhibit equal spatiotemporal correspondences consistent with distinct apparent motion trajectories (i.e., vertical/horizontal) and an at-the-level-of-the-image ambiguous motion signal, unlike the Wandering Circles, the motion observed in these displays tends to be internal to the stimuli. That is to say, the motion occurs at the perceived locations of the stimulus without involving apparent displacements across the visual field beyond these veridical positions. Thus, the Wandering Circles percept may better resemble something akin to the autokinetic effect, by which points presented on a homogenous field (e.g., light spots in darkness, dark spots on white walls) appear to move about in random directions (Ramachandran, Chunharas, Croft, & Batal, 2018; Shapiro & Todorovic, 2016). However, while it appears that both positional uncertainty and lack of reference frame may play a role in creating the autokinetic effect, we can demonstrate (see Video 15) that the illusory motion in the Wandering Circles illusion is largely unhampered, even if a frame of reference such as a stationary box drawn around the circles is introduced.

**Video 15.** Demonstration of typical Wandering Circles illusion with stationary boxes drawn around circles. One may note that the illusion is still present, even when an immediate point of reference, such as the box, is added.

**Figure 7.**
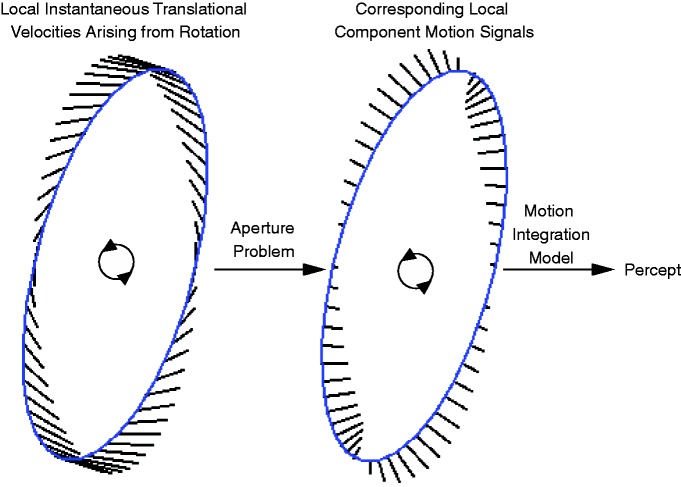
Basic principles of aperture-problem-constrained models of motion perception. Left ellipse: Each location along a moving object’s contour moves with a veridical instantaneous velocity. Here, these velocities are indicated with line segments whose lengths correspond to the ellipse rotating at a constant angular velocity. Many models transform these velocities according to how early-stage motion-detecting neurons constrained by the aperture problem would respond to the moving contour passing through their receptive fields. These component motion signals are illustrated in the right ellipse and generally serve as the inputs into algorithms for describing how the signals may be integrated and ultimately represent how the motion of the object is perceived.

While further research is necessary to fully clarify all of the factors within the stimulus itself (flicker rate, luminance, shape, etc.) that contribute to the perception of illusory motion, a logical next step will be to explore what this phenomenon can reveal about the neural mechanisms of motion perception and the creation/updating of mathematical models for how our visual system handles the perception of visual motion. Currently, it is understood that the earliest stages of the neural processing of visual motion involve the local detection of spatiotemporal changes of luminance or other visual features across the retina (Albright & Stoner, 1995). Many of these locally detected motion signals are one-dimensional and oriented orthogonally to the contour of the moving object (Adelson & Movshon, 1982; Nakayama & Silverman, 1988a, 1988b; Shimojo, Silverman, & Nakayama, 1989). To solve this so-called aperture problem, later stages of neural processing segment and integrate these local motion signals to yield two- or three-dimensional motion signals representing the motion of the object as a whole (Albright & Stoner, 1995; Curran & Braddick, 2000; Heeger, Simoncelli, & Movshon, 1996; Mather & Pavan, 2009; Nishida, 2011; Rust, Mante, Simoncelli, & Movshon, 2006). Over the past several decades, inspired in part by computer vision, powerful computational models have been derived for how this integration process may be implemented in the brain (Adelson & Bergen, 1985; Adelson & Movshon, 1982; Hildreth, 1984; Marr & Ullman, 1981; van Santen & Sperling, 1984; Watson & Ahumada, 1985). As illustrated in Figure 7, at the core of these models is an analysis of local velocities at the level of the stimulus, as detected by neurons constrained by the aperture problem.

That said, the Wandering Circles reveal an important truth about the nature of motion perception and have implications for the way computational models of motion perception are developed and their outputs interpreted. What makes the Wandering Circles important for the study and computational modeling of motion perception is the fact that at the level of the image, there is no net motion information. The contrast-polarity reversals do indeed generate local motion signals, which, in the case of the contours illustrated in Figure 7, would be consistent with expansion and contraction. However, these motion signals are uniformly distributed around the circular contours, equal and opposite in nature, and as such, there is no net directional motion signal, which of course is consistent with the nonmoving stationary nature of the circles. Computational models of motion perception that are based on the detection and integration of motion signals at the level of the image will correctly identify that the Wandering Circles are not physically moving. However, they cannot easily account for the fact that the circles *appear* to move. To do so, such models must incorporate additional constraints that account for the neural mechanisms that lead to the illusory motion observed in the Wandering Circles. Furthermore, while the current study highlights the stimulus features that increase the likelihood of experiencing a motion percept, in related work, we used a similar paradigm to examine interactions between flicker, real motion, and perceived motion and the importance of overall context in determining motion perception (Erlikhman et al., 2019).

In conclusion, under certain circumstances, stationary objects can appear to move. This can be true even in cases where there is no net directional motion energy in the image or in a transformation of the image that takes into account the aperture problem. As such, the illusory motion observed in the Wandering Circles cannot be directly accounted for by a strict analysis of the image. This highlights the necessity for computational models of motion perception that have been classically focused on image-level analysis to ultimately incorporate constraints that account for the multiple sources of heterogeneity that can bias the integration of local motion signals from moment to moment.
